# AGEING WELL WITH AUTISM

**DOI:** 10.1093/geroni/igad104.0135

**Published:** 2023-12-21

**Authors:** Laura Brown, Rebecca Aitken, Katherine Berry, Emma Gowen

**Affiliations:** University of Manchester, Manchester, England, United Kingdom; The University of Manchester, Manchester, England, United Kingdom; University of Manchester, Manchester, England, United Kingdom; University of Manchester, Manchester, England, United Kingdom

## Abstract

Autism is characterized by patterns of neurological development that can cause differences in social interactions and sensory processing; restricted and repetitive patterns of behavior; and difficulties in identifying and describing feelings. The highly sociable and unpredictable environments typical of most societies are poorly suited to autistic people*, leading to disadvantage and exclusion. Despite increasing numbers of people aging with autism, little is known about the needs and experiences of middle-aged and older autistic adults. This is important given that autistic people experience higher rates of health and functional difficulties, and express a need for appropriate, accessible forms of support as they age. In this study, we interviewed seventeen autistic adults, aged from 46-72 years, about their views and experiences of aging, and how services could better support them to age well. The transcribed interviews were analyzed using inductive thematic analysis. The findings revealed several potential ways that autism affected people’s ability to age well. This included difficulties in accessing formal and informal supports; interactions between autism and health conditions; and negative attitudes and low understanding about autism encountered within services and society. Recommendations for supporting autistic adults to age well included involving autistic people in the design of services, and introducing specialized advocate/coordinator roles or ‘one-stop-shop’ hubs that could help autistic people navigate services more easily. * Our use of the terms ‘autistic people/adults’ is in-line with evidence-based guidance for writing about autism research, and is preferred by autistic people to ‘person-first’ terms like ‘person with autism’ (https://journals.sagepub.com/pb-assets/cmscontent/AUT/Autism-terminology-guidance-2021-1626860796.pdf)

